# Human umbilical cord mesenchymal stem cells improve uterine incision healing after cesarean delivery in rats by modulating the TGF-β/Smad signaling pathway

**DOI:** 10.1007/s00404-024-07381-w

**Published:** 2024-02-11

**Authors:** Qing Sun, Dan Zhang, Qiuying Ai, Yang Yue, Haijiao Wang, Le Tang, Xiling Yi, Siyuan Wang, Yang Zheng

**Affiliations:** 1 Postgraduate Training Base of Shenyang Women’s and Children’s Hospital of Jinzhou Medical University, Shenyang, Liaoning, China; 2 Shenyang Women’s and Children’s Hospital, No.87, Danan Street, Shenhe District, Shenyang, Liaoning China; 3 Liaoning Zhongtian Stem Cell and Regenerative Medicine Innovation Research Institute, Liaoning, China

**Keywords:** HU-MSCs, TGF-β/Smad signaling pathway, Cesarean section, Scar healing, Fibrosis, Antifibrosis

## Abstract

**Objective:**

Although human umbilical cord-derived mesenchymal stem cells (HU-MSCs) have attracted increasing attention because of their pivotal functions in the process of wound healing, the underlying molecular mechanisms have been poorly understood. It has been shown that the TGF-β/Smad signaling pathway plays an important role in the process of scar formation. The present study focused on exploring whether HU-MSCs improve uterine incision healing after cesarean delivery in rats via the TGF-β/Smad signaling pathway.

**Study Design:**

Pregnant rats were randomly assigned to three groups, including the NP group, incision-injected group (HU-MSCs1 group), and tail vein-injected group (HU-MSCs2 group), and 30 days after cesarean section, sampling was carried out to further explore the specific mechanisms from tissue and protein levels.

**Results:**

HU-MSCs secretion could inhibit the fibrosis of scar tissue. We observed that the TGF-β induced expression of TGF-β1, Smad2, and Smad3 was attenuated upon HU-MSCs treatment in scar tissue, while the decrease in TGF-β3 expression was enhanced by HU-MSCs. Furthermore, HU-MSCs treatment accelerated wound healing and attenuated collagen deposition in a damaged uterine rat model, leading to the promoting of uterine incision scarring. In addition, the expression of alpha-smooth muscle actin (a-SMA) was enhanced by HU-MSCs treatment.

**Conclusion:**

HU-MSCs transplantation promotes rat cesarean section uterine incision scar healing by modulating the TGF-β/Smad signaling pathway.

## What does this study add to the clinical work?

**Table:** 

HU-MSCs have been used in a variety of fields, but their specific mechanisms for promoting wound healing have not been clarified, especially for uterine incisions, and this paper focuses on exploring the effects of HU-MSCs on the healing of uterine incisions after cesarean section in rats and their mechanisms	

## Background

Cesarean section is one of the most important therapeutic means of addressing difficult labor and high-risk pregnancies, and its rational use is crucial for improving pregnancy outcomes, but it also creates uterine scarring, with attendant complications such as keloid pregnancy and uterine rupture [1]. The uterus provides the internal environment necessary for embryo implantation and pregnancy maintenance, and in women of childbearing age, the normal endometrium undergoes approximately 400 cycles of proliferation, differentiation, shedding, and regeneration without scar formation [2]. The basal layer of the endometrium is permanent and acts primarily through the activity of the endometrium’s intrinsic stem or progenitor cells, which are used to produce a new functional layer in preparation for follicular implantation during each menstrual cycle. Cyclic scarless repair of the endometrium is the only example of scarless repair of adult tissues, a process that involves inflammation, proliferation, and differentiation of endometrium-intrinsic stem cells, and tissue remodeling [3]. When cesarean section causes severe uterine trauma and damages the endometrial fundus, a loss of resident stem cells and stem cell ecological balance occurs, triggering excessive activation of fibroblasts and continuous collagen secretion, and excessive collagen deposition prevents proliferation, differentiation, and migration of the natural cells of the uterus, so that the damaged uterine wall, and in particular, the endometrium, ultimately develops collagenous scarring [4].

Mesenchymal stem cells (MSCs) are pluripotent stem cells with unique biological potential, and their therapeutic potential is attributed to the ability of the multiplex lineage to differentiate, self-renew, and secrete a wide range of paracrine factors, such as growth factors, cytokines, and chemokines, via secretion to their surroundings [5]. These paracrine factors have anti-scarring, supportive, and angiogenic functions and are the most biologically active components in the reconstruction of damaged tissues induced by MSCs [6]. Human umbilical cord-derived mesenchymal stem cells (HU-MSCs) as a primitive population of MSCs between fetal and adult MSCs, have shown outstanding advantages in terms of abundant supply, painless collection, low immunogenicity, and high proliferative potential [7, 8]. Xin et al. found that HU-MSCs can promote the proliferation of human endometrial stromal cells and inhibit apoptosis through paracrine effects to increase the expression levels of estrogen receptor α and progesterone receptor and improve fertility [9]. Due to the emerging role of HU-MSCs in uterine repair, transplantation of HU-MSCs has become a common way of studying uterine tissue regeneration [10, 11].

TGF-β is considered to be an important fibrogenic factor, and the typical signaling pathway of TGF-β involves the downstream Smad molecule [12]. The binding of TGF-β to its family of receptors (serine/threonine kinase cell-surface receptors) leads to direct carboxyl-terminal phosphorylation of Smad2 and Smad3, which translocate to the nucleus in a complex with Co-Smads, and then activate the TGF-β/Smad signaling pathway. Smad signaling pathway, inducing excessive proliferation of fibroblasts and excessive deposition of extracellular matrix (ECM), leading to tissue fibrosis and scar formation, in which TGF-β1 is positively correlated with scar formation, while TGF-β3 inhibits pathologic scar formation [13]. However, the relationship between TGF-β1, TGF-β3, and TGF-β/Smad signaling pathways HU-MSCs is unclear and requires further study.

In this study, we constructed a rat model of cesarean delivery, detected the expression of relevant factors in the TGF-β/Smad signaling pathway, and explored whether HU-MSCs were promoting uterine incision healing by regulating the TGF-β/Smad signaling pathway.

## Materials and methods

### Experimental materials

Clean-grade adult healthy female and male SD rats were selected, weighing 220–250 g for females and 250–300 g for males, provided by the Laboratory Animal Center of the General Hospital of the Northern Theater of Operations, and housed in an environment with a room temperature of 22–25 ℃ and good ventilation and lighting conditions; the rats were fed in special cages with special feed and water. All animal experiments were conducted in accordance with the guidelines and research protocols of the Ethics Committee for Animal Experiments of the Chinese Academy of Agricultural Sciences, and the study was approved by the Ethics Committee of Shenyang Women’s and Children′s Hospital, and the HU-MSCs were extracted in cooperation with Liaoning Zhongtian Stem Cells and Regenerative Medicine Innovation Research Institute Co.

## Animal experiments

Cages were combined according to female: male = 2:1, and the following morning at 8:00 a.m. vaginal secretions were taken for microscopic examination, and spermatozoa were found on day 0 of gestation. On the 20th day of gestation, the rats were randomly divided into three groups for cesarean section after anesthesia, which were divided into a NP group (*n* = 7), HU-MSCs1 group (*n* = 7), and HU-MSCs2 group (*n* = 7). The procedure of cesarean section was as follows: a 3.0-cm incision was made in the lower abdomen of the rat, a 1.5-cm longitudinal incision was made in the middle of the uterine horns on both sides along the contralateral side of the mesentery, and the fetus and the placenta were gently squeezed through the uterine incision and removed, and the ends of the uterine incision were marked with 6-0 silk sutures, and the uterine incision was closed by successive suturing of 6-0 absorbable sutures, in the control group, the ends of the uterine incision were labeled with 6-0 silk sutures, and the uterine incision was closed continuously with 6-0 absorbable sutures. In the HU-MSCs1 group, after the ends of the uterine incision were labeled with 6-0 silk sutures and the uterine incision was closed continuously with 6-0 absorbable sutures, the uterine incision was injected directly into the myometrium on both sides of the uterine incision in five spots, and each spot was grafted with 1 × 10^6^ HU-MSCs in 50 μL PBS; In the HU-MSCs2 group, the ends of the uterine incision were marked with 6-0 silk sutures, and after the uterine incision was closed with 6-0 absorbable sutures in a continuous fashion, 1 × 10^6^ HU-MSCs in 250 μL of PBS were injected into the tail vein. The fascia and skin were closed continuously with 4-0 absorbable sutures. The rats were executed by euthanasia 30 days after cesarean section, and tissue specimens from the transverse uterine incision and serum specimens were obtained.

## Culture and characterization of HU-MSCs

HU-MSCs were obtained from Zhongtian Stem Cell and Regenerative Medicine Innovation Research Institute Co, Liaoning, China, and at the same time, they confirmed the identity of the HU-MSCs using a flow cytometric method using a panel of recognized HU-MSCs surface markers, including positive expression for CD73, CD90, CD105 and negative expression for HLA-DR, CD79a, CD14, CD19, CD34, CD45. The HU-MSCs were cultured in Dulbecco’s Modified Eagle Medium/Ham’s F-12 (DMEM/F12) supplemented with 20% fetal bovine serum (FBS), 5 ng/ml epidermal growth factor, 100 U/ml penicillin and 100 mg/ml streptomycin in an incubator (37 °C, 5% CO_2_). The initial design of this experiment aimed to transplant adenovirus-transfected stem cells. However, due to various reasons, the transplantation of adenovirus-transfected stem cells did not occur as planned. The 5th–6th generation cells were used for the experiment [14].

## Histological analysis

Uterine specimens were collected on the 30th day after cesarean section. The specimens were fixed in 4% paraformaldehyde, dehydrated in graded alcohol, cleared in xylene, and finally paraffin-embedded. The tissue was cut crosswise at a thickness of 5 μm. The morphological structure of the uterus was evaluated by HE staining, and the thickness of the smooth muscle in the uterine scar area, and the vascular density was assessed by the number of capillaries counted from six randomly selected areas in each region at a magnification of × 400. Collagen deposition around the uterine scar was observed by Masson trichrome staining.

## Immunohistochemical analysis

The expression of anti-α-smooth muscle actin (α-SMA) was detected by immunohistochemistry in uterine incision tissues. Sections were routinely deparaffinized, placed in distilled water, rinsed under running water for 1 min and then stained with Weigert iron hematoxylin A and B solution mixed 1:1 for 5–10 min, washed in water, and titrated with α-SMA primary antibody (the antibody; Servicebio. China) at a dilution of 1:2000, and incubated overnight at 4 °C, rewarm at room temperature for 1 min, rinse with PBS and develop color. After re-staining, dehydration, and sealing, permanent slide specimens were made for observation. α-SMA immunohistochemical staining of positive cells as brownish-yellow granules. The percentage of α-SMA-positive area × 6 areas was randomly selected at 100 × magnification, and the ratio of the α-SMA-positive area to the total area of the muscle layer of the selected areas was calculated by Image-Pro Plus software, and the mean value was taken.

## Real-time PCR analysis

Real-time PCR was applied to detect TGF-β1, and TGF-β3 in the uterine incision tissues, Trizol reagent was applied to collect total RNA in the uterine incision tissues, and ABI kit (Servicebio. China) and SYBR Green Master Mixed Set real-time PCR was applied to detect cDNA reverse transcription products. Relevant primers were acquired from Invitrogen (Table 1). To quantify these reagents, β-actin gene normalization was applied. Gene amplification was analyzed using the 2-ΔCt algorithm [15].

**Table 1:** The primers of Real-time PCR

Name	Primer	Sequence(50–30)
Rat TGF-β1	Forward	AGAGCCCTGGATACCAACTA
	Reverse	CAACCCCAGGTCCTTCCTAAAG
Rat TGF-β3	Forward	CACTGTCCATGTCATACCT
	Reverse	CTTCGTTGTCCACTCCTT

## Western blot analysis

Western blot was applied to detect the protein expression of Smad2 and Smad3. The uterine incision tissues of each group were removed from a −80 ℃ refrigerator, cut and weighed 20 mg of tissues, transferred to a homogenizer with liquid nitrogen, and fully milled; Add 1 ml RIPA lysate [containing 10 μl phenylmethylsulfonyl fluoride (PMSF)] to the EP tube after tissue milling, lysing the tissue for 30 min, extracting the total protein, and determining the protein concentration with the BCA Protein Assay Kit (Servicebio. China), adding 1/4 sample volume amount of the uploading buffer after the assay, and then 100 ℃ metal bath for 10 min, and then standby at room temperature. Adjust the electrophoresis parameters. After removing the gel, the membrane was transferred by the wet transfer method, and the membrane was closed with skimmed milk for 2 h. Anti-Smad2, anti-Smad3 antibody, and anti-β-actin antibody (Servicebio. China) (dilution ratio of 1:3000) were added respectively, and the membrane was shaken at 4 ℃ overnight. Add IgG antibody (Servicebio. China) (dilution ratio of 1:3000) and incubate at 37 ℃ for 1 h. Luminescence development was performed with ECL Plus luminescent solution, and gray scale analysis was performed by gel image analysis system, and the gray scale value of each protein band was determined by Image J 1.8.0 image analysis system, and the relative expression of Smad2 and Smad3 proteins was calculated by using β-actin as the internal reference.

## Statistical analysis

The data obtained were processed by SPSS 27.0 statistical analysis software, and the resulting specimens were subjected to single-blind image acquisition and histological analysis, expressed as mean ± standard deviation (SD), and analyzed by t-test or analysis of variance for multiple comparisons. *P* < 0.05 differences were statistically significant.

## Results

### Identification and characterization of isolated HU-MSCs

**Fig. 1:**
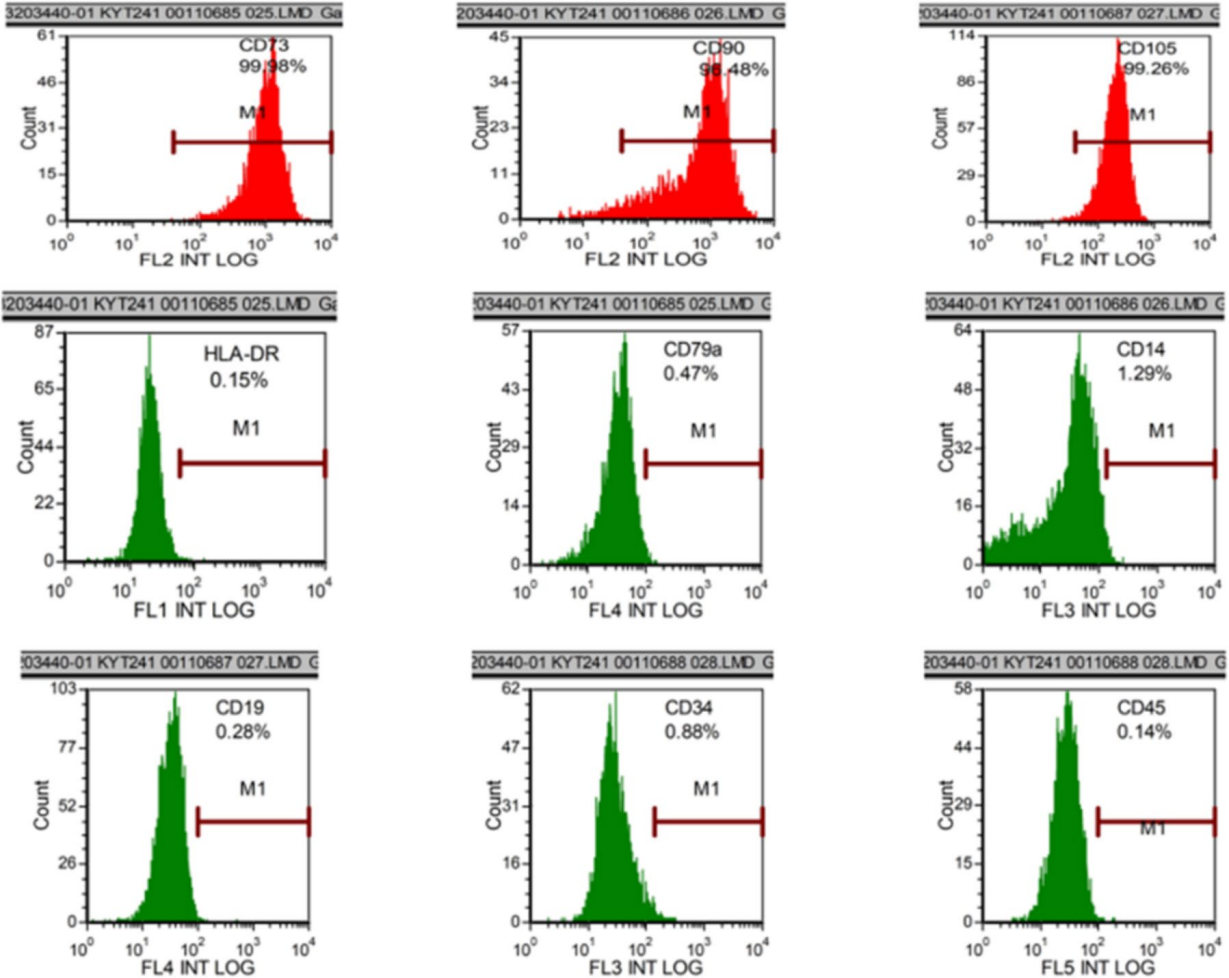
Identification and characterization of isolated HU-MSCs

Flow assay for cell surface markers of HU-MSCs showed that HU-MSCs highly expressed CD73, CD90, CD105, and low or no expression of HLA-DR, CD79a, CD14, CD19, CD34, CD45 (Fig. 1).

### HU-MSCs transplantation improves uterine tissue morphology after cesarean delivery in rats

**Fig. 2 (:**
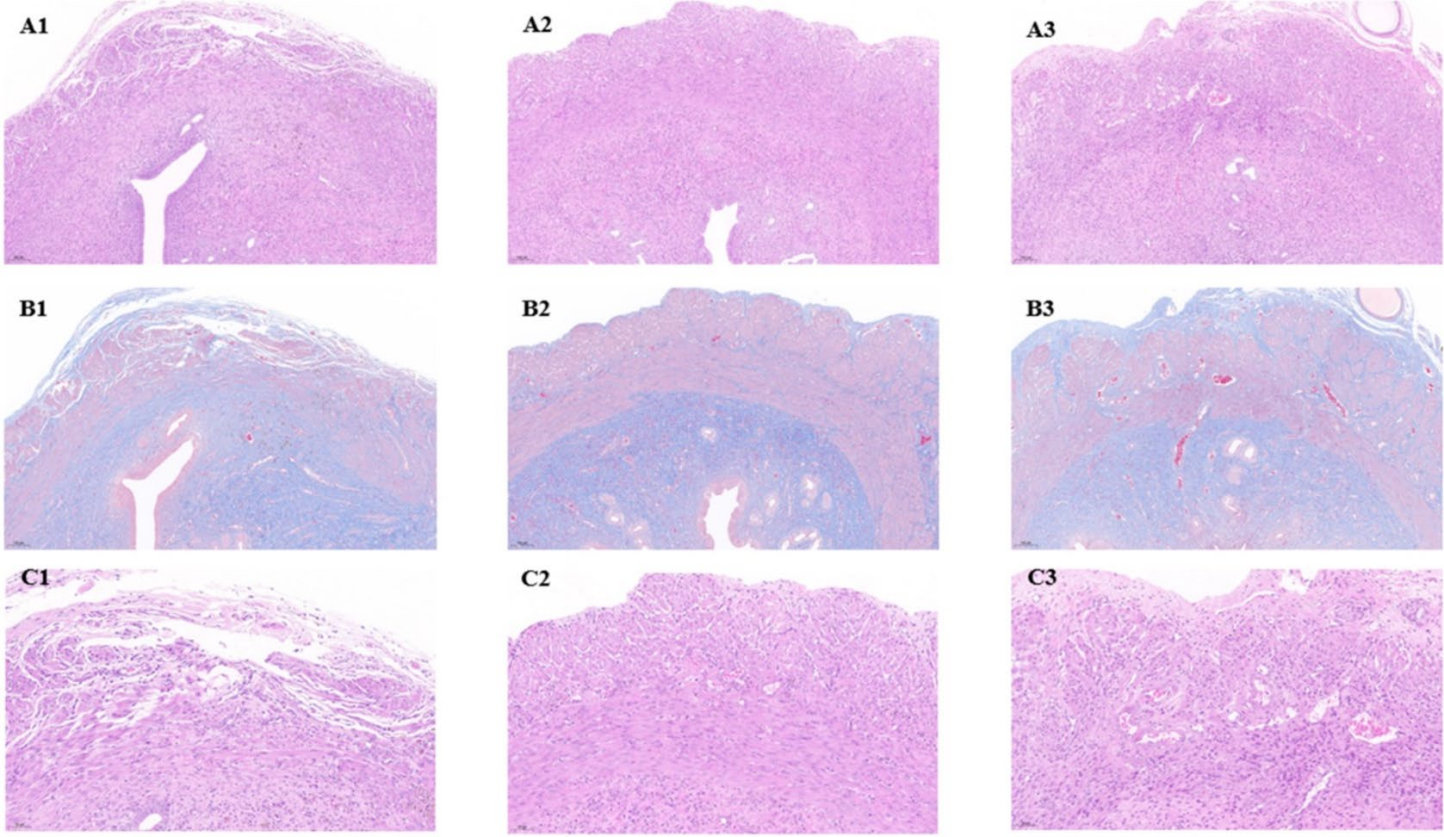
**A**, **C**): HE; (**B**): Masson; 1: NP group; 2: HU-MSCs 1 group; 3: HU-MSCs 2 group; (**A**, **B**): × 100; C: × 200

The recovery of myometrial tissue is an important observation index of uterine scar regeneration, according to HE staining, we observed that on the 30th day after cesarean section, no obvious capillaries were seen in the scar area of the control group, the myometrium was thin and disordered, the endothelium was thin, and the epithelium was interrupted and discontinuous in severe cases, with a large number of inflammatory cells infiltrating, and even mesenchymal hyperplasia and edema, and the formation of granulation tissue. There was no significant difference between the conditions of HU-MSCs 1 and 2 groups, both of which could see newborn abundant capillaries, well-organized muscle bundles with new independent muscle layers, which were similar to the normal tissues, muscle fibers were neatly arranged, muscle layer and endothelial thickness were thicker than those of the control group and orderly distributed, and the endothelial interruption was improved, and the inflammatory cell infiltration was significantly reduced (Fig. 2).

Collagen deposition is an essential feature common to all scarring, and its presence prevents the proliferation, differentiation, and migration of the natural cells of the uterus. The extent of collagen deposition in the uterine scar was assessed by Masson trichrome staining. On the 30 days after cesarean section, the uterine scar in the HU-MSCs 1 and HU-MSCs 2 groups showed significant collagen degradation and increased muscle bundles compared with the control group, and there was no significant difference between the two groups (Fig. 2).

### HU-MSCs transplantation promotes myometrial healing after cesarean delivery in rats

**Fig. 3:**
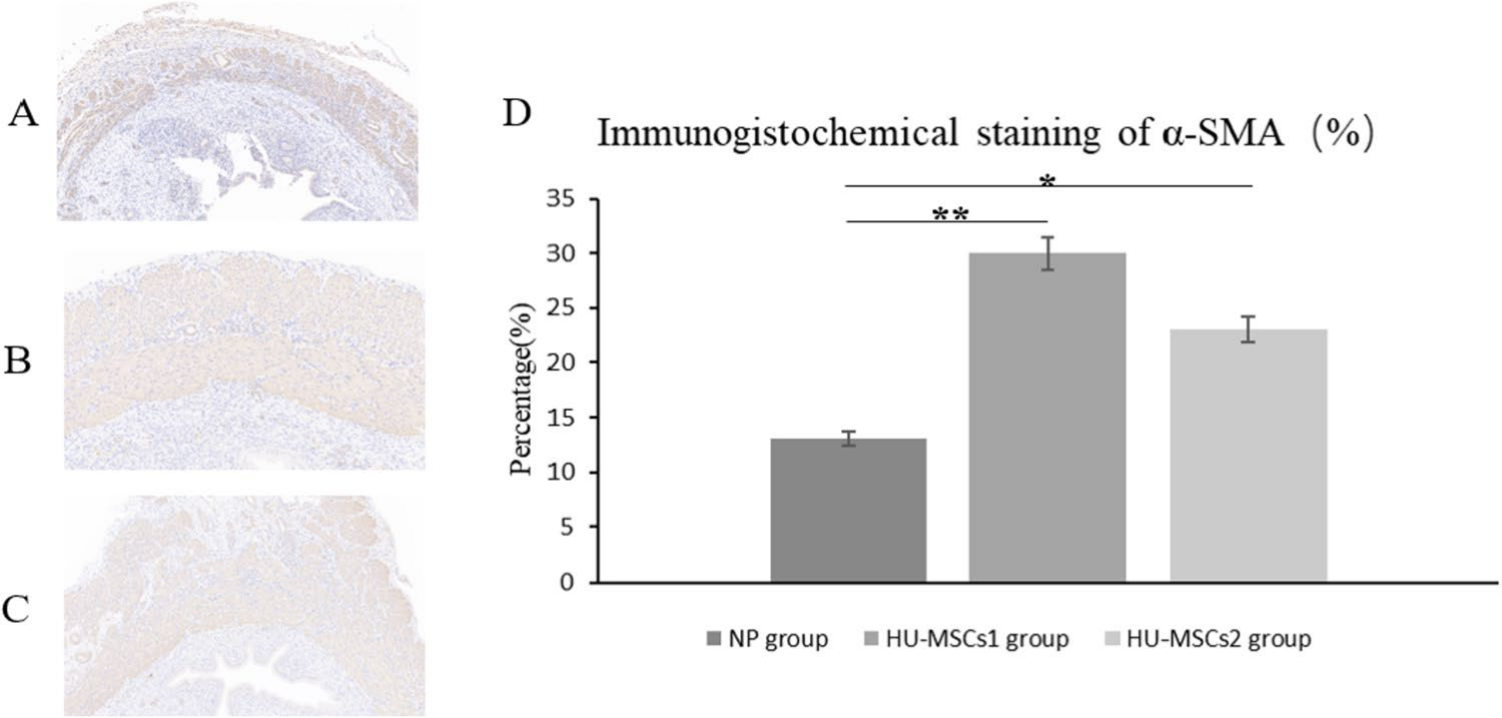
The α-SMA expression of the uterine incision tissue was observed by immunohistochemical staining. (**A**) is the control group; (**B**) is the HU-MSCs 1 group; (**C**) is the HU-MSCs 2 group. (**D**) is Immunohistochemical staining of α-SMA. Data are expressed as mean values. *P < 0.05 and **P < 0.01

On the 30th day after cesarean section, a significant increase in the expression of α-SMA in the HU-MSCs 1 group and the HU-MSCs 2 group could be observed, and the expression of α-SMA in the control group was less. The percentages of α-SMA positive regions in the HU-MSCs 1 and HU-MSCs 2 groups were (30.14% ± 6.64%) (22.71% ± 7.09%), respectively, which were significantly higher than those in the control group (12.71% ± 2.81%), and the differences were statistically significant (Fig. 3), where there was no obvious difference.

### HU-MSCs transplantation modulates TGF-β mRNA expression in the TGF-β/Smad signaling pathway

**Fig. 4:**
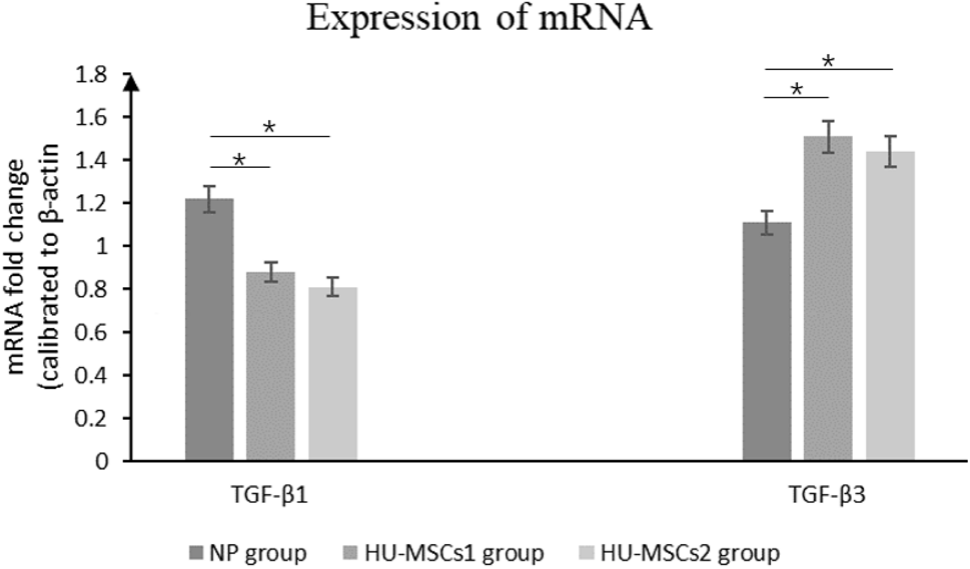
Factors secreted from BMSCs alleviate TGF-β/Smad signaling. RT-qPCR detection of mRNA expression of components in TGF-b/Smad signaling

TGF-β is a cytokine that influences cell growth and differentiation and is essential in wound healing and scar formation. To further explore the relationship between HU-MSCs and TGF-β in these processes, we examined the mRNA expression of TGF-β1 and TGF-β3 in uterine incision tissues using real-time PCR. Compared with the control group, the mRNA level of TGF-β1 was significantly lower in both HU-MSCs 1 and HU-MSCs 2 group, while TGF-β3 was significantly higher in HU-MSCs 1 and HU-MSCs 2 group than the control group, with a specific statistically significant difference, whereas there was no significant difference in the expression of mRNA of TGF-β1 and mRNA of TGF-β3 between the two groups when comparing the HU-MSCs 1 and HU-MSCs 2 group (Fig. 4).

### HU-MSCs transplantation modulates SMAD protein expression in the TGF-β/Smad signaling pathway

**Fig. 5:**
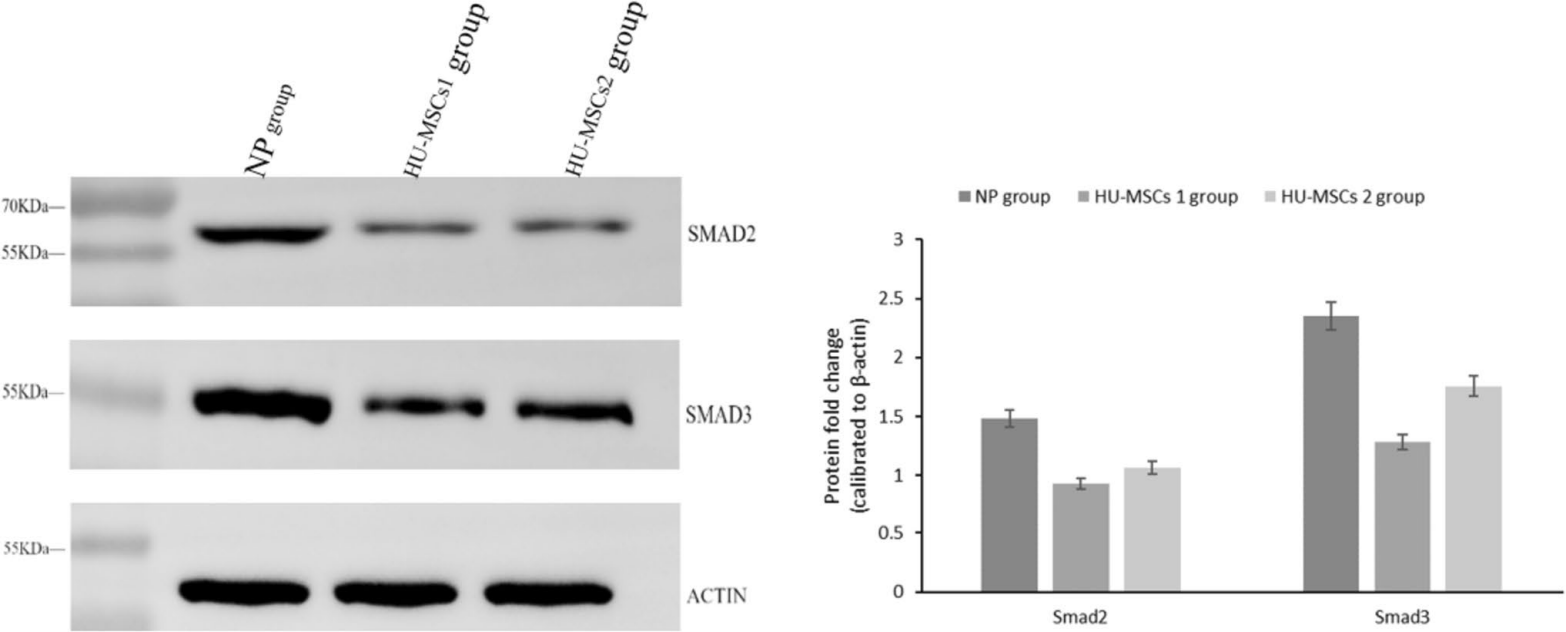
Representative Western blot results and quantification of protein expression of components in TGF-β/Smad signaling

In the TGF-β/Smad signaling pathway, Smad2 and Smad3 are key molecules that bind to TGF-β. We used protein blotting to detect their protein expression levels. The results showed that Smad2 and Smad3 protein expression was significantly reduced in both the HU-MSCs 1 and HU-MSCs 2 group compared with the control group, and the difference was statistically significant; Smad2 and Smad3 protein expression of TGF-β1 in the HU-MSCs 1 group compared with the HU-MSCs 2 group, and there was no significant difference between the two groups (Fig. 5).

## Discussion

In recent years, MSCs have demonstrated great application prospects, and several studies have now shown that they play the roles of self-renewal, multilineage differentiation, anti-inflammation, and promotion of regeneration of damaged tissues through secretion of various cytokines [16–18]. Recent studies have identified the roles of MSCs in repairing tissue damage and promoting tissue regeneration through paracrine secretion [19, 20]. As a type of perinatal stem cells, HU-MSCs are anti-rejection and can be used for cell transplantation without histocompatibility antigen mating [21]. A large number of MSCs can be obtained from the umbilical cord, which is safe and convenient, and overcomes the shortcomings of bone marrow MSCs whose number and proliferative and differentiation potentials decline with age, and which have a high rate of viral infections, allowing for a wide range of clinical applications [22, 23]. Scarred uterus as one of the most common adverse effects after cesarean section, collagen fibrin deposition is one of the main problems, which affects cell proliferation, differentiation, and migration, which is mainly due to fibroblast proliferation and overproduction of ECM [24]. Most of the current treatments are aimed at the prevention of uterine adhesions and intra-abdominal adhesions in the relevant organs [25], and most studies on the molecular signaling and cellular mechanisms of fibrosis in various organs have focused on the liver, kidney, lung, heart, or skin [26]. There are only a few studies have been directed at intervening in the hyperfibrosis and collagen deposition of uterine incisions, and the molecular mechanism of the facilitating effect of transplanted HU-mesenchymal stem cells on uterine incision healing has also been a major challenge.

The application of HU-MSCs was shown by HE and Masson trichrome staining to effectively attenuate collagen deposition in wound-healing tissues and promote uterine incision healing after cesarean section. The results are consistent with the study of Li et al. who established a rat uterine whole-layer scar model and found that HU-MSCs on scaffolds promoted collagen deposition and regeneration of endometrium, myometrium, and blood vessels in a rat uterine scar model [27]. However, Xu et al. showed [4] that HU-MSCs promote collagen degradation and regeneration of endometrium, myometrium, and blood vessels in uterine scarring by upregulating MMP-9.

The integrity of the uterus is crucial for embryo implantation and pregnancy maintenance, and in a study by Buhimschi [28] and others, uterine tissue injury must be restored to its original cellular and tissue structure to regain its own function. Kuramoto G et al. demonstrated that transplantation of HU-MSCs sheets was feasible in a rat model of hysterectomy and showed that stem cell sheets transplanted into hysterectomies promoted myometrial regeneration and reduced fibrotic tissue formation [29]. The rat uterus histologically shows a tubular lumen, with an outer membrane, myometrium, and endometrium in that order from the outside to the inside. Transparent plasma membrane layer for the outer membrane, thin as paper, and the muscle layer adheres closely, not easy to separate; by the inner ring of the outer longitudinal composition of the two layers of smooth muscle for the myotome, thicker, with the ability to contract, myocytes are arranged in an orderly manner, within the blood vessels can be seen, loose connective tissue; epithelium and mesenchyme composed of the endothelium, the epithelium is a tightly arranged monolayer of columnar epithelium, in the form of a pleat, and the mesenchymal stroma contains a single tubular glands. In this experiment, we initially explored the repairing effect of HU-MSCs on uterine incision scar in rats by 2 modes of local myometrial multipoint injection and tail vein injection. The results showed that compared with the HU-MSCs 1 and HU-MSCs 2 group, the myometrial layer in the uterine scar region of the control group was thinned, with myofibrillar breaks, or even defects, and increased collagen fibers; the endothelial layer was thinned, with severe epithelial defects; myometrial thickness, endometrial thickness, and percentage of α-SMA-positive area were lower than those in the HU-MSCs 1 and HU-MSCs 2 groups, and the rate of endometrial defects was higher than those in the HU-MSCs 1 and HU-MSCs 2 groups, which proved that the HU-MSCs contributed to the growth of the uterine smooth muscle cells, and facilitated the restoration of the original structure of the site, which was in agreement with the findings of Fan Y et al. [30]. When comparing the HU-MSCs 1 and HU-MSCs 2 groups, the differences in myometrial thickness and the percentage of the α-SMA-positive area were not statistically significant, suggesting that both methods of administration can repair uterine incision scarring.

Together with α-SMA, TGF-β is a key cytokine in regulating ECM metabolism and initiating the fibrosis process. It promotes the synthesis of fibronectin and the proliferation of interstitial cells through the regulation of fibroblast differentiation and inhibits the breakdown of ECM, which ultimately leads to the development of fibrosis [31, 32]. TGF-β1, in particular, is considered to be one of the most active fibrotic cytokines. High levels of TGF-β1, a powerful driver of myofibroblast differentiation, are highly associated with scar formation [32, 33]. To further investigate whether the incision scar healing-promoting effect of HU-MSCs was related to the TGF-β/Smad signaling pathway, we examined the mRNA and protein expression levels of the key molecules involved in this signaling pathway, such as TGF-β1, TGF-β3, Smad2, and Smad3, and the results showed that, compared with the control group, the mRNA and protein expression levels of TGFβ1, Smad2, and Smad3 were significantly reduced, except TGF-β3, in both the HU-MSCs 1 group and HU-MSCs 2 group, this is consistent with the results of related studies [32, 34]. It provides an effective molecular basis for further study of the mechanism of HU-MSCs in promoting uterine incision healing.

In summary, these in vivo data demonstrate the therapeutic effect of HU-MSCs on scar formation by enhancing the wound healing process. It is suggested that HU-MSCs attenuate tissue fibrosis and inhibit collagen deposition in vivo, which may be related to the TGF-β/Smad signaling pathway.

## Conclusion

In this study, we demonstrated in a rat model that HU-MSCs transplantation may attenuate scar tissue fibrosis, reduce collagen deposition in uterine scars, and promote regeneration of myometrium and vasculature in uterine scars by modulating the TGF-β/SMAD signaling pathway. From the perspective of structural reconstruction, uterine scars treated with HU-MSCs transplantation improved uterine scar outcomes. Overall, HU-MSCs transplantation may be a novel therapy for uterine scars. However, the TGF-β family consists of a number of structurally related differentiation factors that act through cell-surface heterodimeric receptor complexes and intracellular signaling Smad complexes [35]. The drawbacks of this study are that it was not possible to exclude cellular interference and that the cells themselves are involved in attenuating the process of scarring and collagen deposition by a variety of complex mechanisms. The mechanisms are diverse and complex. Therefore, further studies are needed to elucidate the mechanism of action of HU-MSC-mediated uterine scar formation and attenuated collagen deposition.

This study also demonstrates the potential of HU-MSC transplantation in treating uterine scars. If these experimental results can be validated in human clinical practice, HU-MSC transplantation may become an innovative treatment option, providing more effective treatment for women with uterine scars. This may improve patients’ quality of life, and reduce complications.

However, further clinical research is needed to validate these experimental results and evaluate the safety and efficacy of HU-MSC transplantation in humans. Such research will help determine the optimal use of this treatment method in clinical applications and lay the foundation for future clinical trials.

## Data Availability

The data that support the findings of this study are available from the corresponding author, [Dan Zhang], upon reasonable request.
